# Pattern of Preferred Cataract Surgery Practices in Morocco: A Survey-Based Study

**DOI:** 10.7759/cureus.55690

**Published:** 2024-03-06

**Authors:** Hamza Lazaar, Meryem Sefrioui, Taha Boutaj, Boutayna Azarkan, Rim El Hachimi, Saad Benchekroun, Abdellah Amazouzi, Lalla Ouafa Cherkaoui

**Affiliations:** 1 Ophthalmology A, Hopital Des Specialités, Faculté de Médecine et de Pharmacie de Rabat, Rabat, MAR

**Keywords:** cataract, survey, techniques, phacoemulsification, knowledge

## Abstract

Aim and methodology

The aim of the study was to describe the preferred cataract surgery practices among Moroccan ophthalmologists and compare them with practices in other countries. An online survey consisting of 29 multiple-choice questions was sent to Moroccan ophthalmologists. The questions were centered on the preferred cataract surgical practices of the participants. All the data obtained were collected and analyzed.

Results

A total of 276 participants responded to the survey. Of these, 178 (64,50%) were in the age group of 31-50 years. The visual acuity for which the operative indication was made was 4/10 for 144 (52.4%) participants). The most popular type of anesthesia was topical, reported by 172 (62.4%). Stop-and-chop was the most used technique for routine cataract surgeries, while hydroprolapse of the nucleus was the leading technique for soft cataracts. The two measures are considered crucial for postoperative endophthalmitis prophylaxis: Povidone-iodine instillation into the conjunctival sac and intracameral antibiotics were performed by 267 (97%) and 276 (100%) participants, respectively. Nonsteroidal anti-inflammatory drugs were prescribed by only 128 (46.5%) surgeons.

Conclusion

This study provides some insight into the present cataract surgery techniques in Morocco, which might differ considerably from one country to another. Studies in various countries need to be undertaken to develop a consensus and reach some evidence-based patterns. This study may serve as a guide for young surgeons starting their careers based on what the standard procedures are among their seniors and peers.

## Introduction

Cataract is the leading cause of blindness worldwide [1], and cataract extraction is the most prevalent surgical procedure among all medical specialties [2].

Phacoemulsification with intraocular lens implantation has become the gold standard for cataract surgery today, providing excellent anatomical and functional outcomes, quicker recovery, and lower complication rates, ensuring enhanced safety. Although this surgery is extremely standardized, preoperative and postoperative practices and perioperative surgical techniques can vary from one country to another, from one training center to another, and even from one surgeon to another.

The purpose of our study is to report the results of a national survey conducted in Morocco involving 276 ophthalmic surgeons regarding the preoperative, intraoperative, and postoperative surgical techniques and practices related to cataract surgery and compare them with practices in other countries.

## Materials and methods

A descriptive cross-sectional study was conducted between September 2023 and December 2023 involving ophthalmologists within the Kingdom of Morocco. Ophthalmologists performing cataract surgery who agreed to participate in the study were included. Young residents, participants who did not give their consent, and ophthalmologists who didn't perform cataract surgery were excluded from the study. No personal information was asked and the survey was completely anonymous. It took approximately 15 minutes to complete, with no compensation for the participants.

An online survey consisting of 29 multiple-choice questions regarding cataract surgical practices and habits of phacoemulsification was distributed electronically using Google Forms (Google LLC, Mountain View, California, United States) via a WhatsApp group (Meta Platforms, Inc., Menlo Park, California, United States) of Moroccan ophthalmologists and emails from the Moroccan Society of Ophthalmology.

The data collected included four major sections. The first section focused on demographic and professional information, including age, gender, and work sector. The second part explored preoperative habits, such as the systematic use of specular microscopy, the visual acuity threshold for surgical indication, the type of anesthesia, and safety precautions such as venous access, continuous electrocardiogram monitoring, and pressure monitoring. The third part examined perioperative habits, including the preferred machine system, injection of vision blue, direction of the capsulorhexis, techniques used for routine and soft cataracts, type of intraocular lens, as well as infectious prophylaxis. Finally, the fourth section explored postoperative habits, including postoperative treatment, duration of patch use, time frame for optical correction, and the interval before operating on the other eye.

IBM SPSS Statistics for Windows, Version 26.0 (Released 2019; IBM Corp., Armonk, New York, United States) was used for statistical analysis.

## Results

A total of 276 respondents participated in the study. Table 1 represents the demographic characteristics of the participants. Of the total, 153 (55.43%) were male and 123 (44.57%) were female. Forty-two (15.21%) participants were under 30, 178 (64.50%) were between 30 and 50 years of age and 56 (44.56%) were over 50; 123 (44.57%) worked in the private sector, 89 (32.24%) in the public sector, and 64 (23.2%) in a university hospital.

**Table 1:** Demographic information of surgeons

Variable		Frequency (n)	Percentage (%)
Gender	Male	153	55.43%
	Female	123	44.57%
Age	<30	42	15.21%
	30-50	178	64.50%
	>50	56	20.29%
Sector	Private	123	44.56%
	Public	89	32.24%
	University Hospital	64	23.20%

Preoperative habits

Specular microscopy was systematic only for 60 (21.6%) ophthalmologists. The visual acuity for which operative indication was made was as follows: 4/10 for 145 (52.4%) ophthalmologists, 5/10 for 58 (21%), and for 41 (15%), it was the reported occasional inconvenience regardless of visual acuity.

The leading anesthesia type was topical for 172 (62.4%) respondents, followed by peribulbar block for 58 (21%), subtenon anesthesia for 31 (11.4%), and finally retrobulbar block for 14 (5.2%) surgeons. Venous access was reported by 260 (94.4%) of the respondents, pressure monitoring by 253 (91.6%), and 211 (76.6%) surgeons got electrocardiogram monitoring done.

Perioperative habits

**Figure 1:**
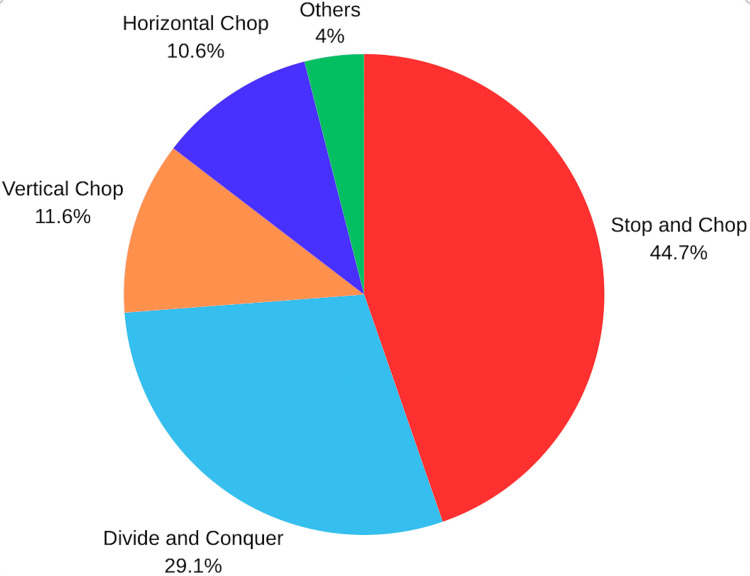
Phacoemulsification technique for routine cases among the participants

Povidone-iodine was instilled in the conjunctival sac before cataract surgery by 265 (96%) of the surgeons. One hundred and sixty-seven (60.4%) surgeons preferred peristaltic systems over venturi pumps 109 (39.6%). The blue vision was systematic for 178 (64.5%) of surgeons. Capsulorhexis was performed in a clockwise direction by 155 (56.4%) ophthalmologists and counterclockwise by 52 (19.1%), while it did not matter for the remaining 69 (24.9%). For routine cases, Stop and Chop was the most commonly used technique (Figure 1).

**Figure 2:**
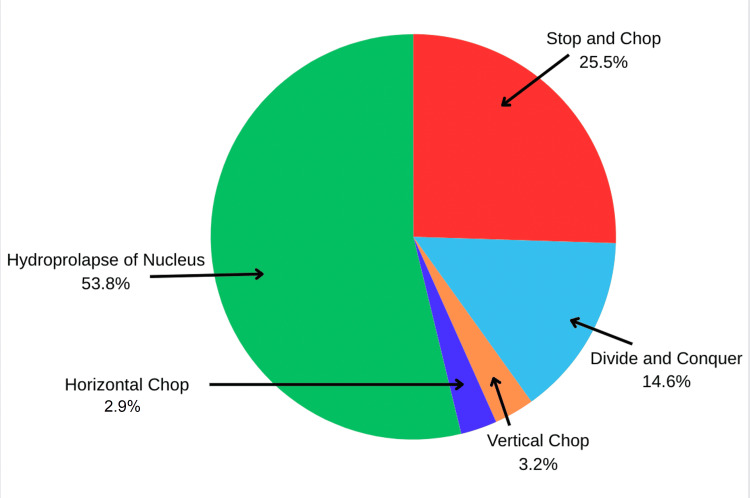
Phacoemulsification technique for soft cataracts among the participants

For soft cataracts, hydroprolapse of the nucleus was the main technique used for this type of cataract (Figure 2).

**Figure 3:**
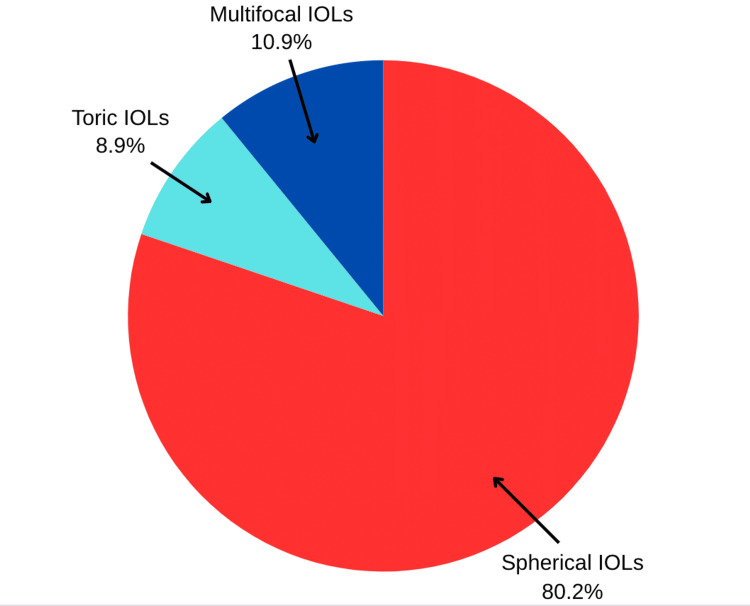
: Intraocular lenses (IOLs) used by the participants

Spherical intraocular lenses were the leading intraocular lenses (IOLs) used (Figure 3).

Hydration of paracentesis was systematic for 253 (92% of surgeons) and prophylactic intracameral antibiotics were used by all the 276 respondents (100%).

Postoperative habits

**Figure 4:**
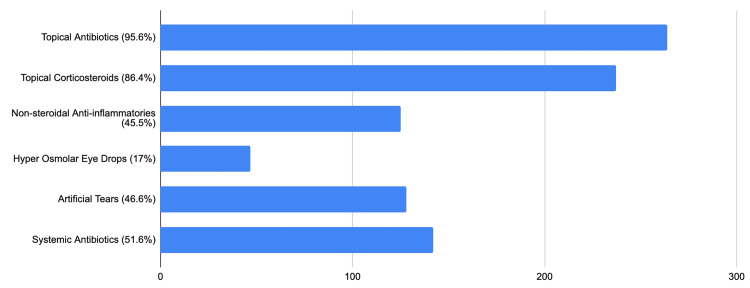
The post-operative treatments prescribed by the participants

The postoperative treatment is illustrated in Figure 4. The ocular patch is removed within two to seven days after surgery by 168 (60.7%) surgeons, after 24 hours post surgery by 42 (15.3%), and after two weeks by 67 (24.3%). The refractive errors remaining after surgery were corrected after one month by 200 (72.8%) surgeons. The second eye is operated on within one month by 139 (50.3%) surgeons), within 15 days by 38 (13.7%), after three months by 35 (12.7%), and 48 (17.3%) did it based on the patient's will.

## Discussion

The preferred cataract surgery procedures currently used by Moroccan ophthalmologists are compiled in this survey.

The sole use of topical anesthesia was the most commonly practiced anesthetic method by 172 (62.5%) respondents, slightly higher than the Malaysian study with 42.8% [3] and lower than the Korean study with over 80% [4]. In comparison to the venturi system, the peristaltic pump was more widely preferred; this preference was similar to the Malaysian study, with greater popularity observed for the peristaltic pump (56,1%) [3].

Trypan blue dye, a staining agent, has demonstrated Level III evidence supporting its effectiveness in staining the lens. Additionally, there is Level II evidence suggesting that staining the capsule with trypan blue is beneficial, particularly in facilitating the completion of capsulorhexis, especially in cases of white cataracts [5]. Vision Blue is systematically used regardless of the type of cataract by 178 (64.7%) respondents, similar to the Malaysian study with 70.1% [3].

Peristaltic pumps use rollers to create flow by compressing the tube and moving the fluid in one direction. A vacuum is formed depending on flow restriction, and the maximum preset vacuum levels can only be achieved with full occlusion of the tip. Venturi pumps use a vacuum to create flow [6]. No study has demonstrated the superiority of one system over the other in terms of safety [7]. In comparison to the Venturi system, the peristaltic pump was more widely preferred; this preference was similar to the Malaysian study, with greater popularity observed for the peristaltic pump too (56,1%) [3]. 

The superiority of one surgical procedure over another remains controversial. Indeed, several studies reveal no significant difference between stop and chop and phaco chop [8], nor between the latter and divide and conquer [9]. However, some studies have demonstrated that, for hard cataracts, the phaco-chop approach may minimize intraoperative parameters and postoperative endothelial cell count loss more efficiently than the stop-and-chop and divide-and-conquer procedures [10]. In our survey, stop and chop was the most used technique for routine cataract surgeries, followed by divide and conquer, and finally direct chop. This was similar to the Malaysian survey [3]. However, in a study describing the habits of French ophthalmic surgeons, divide and conquer was the most popular technique [11].

Hydration of paracentesis was performed systematically by 254 (92%) surgeons in the present study, whereas only 75% of surgeons in the French survey incorporated it in their regular cases [11].

Two measures are considered crucial for postoperative endophthalmitis prophylaxis: (i) Instillation of povidone-iodine 5% into the conjunctival sac as done by 265 (96%) surgeons in the current study, enabling the destruction of 97% of bacteria within one minute [12], and (ii) Intracameral antibiotic injections (performed by 100% of surgeons in the current study) reduce the risk of endophthalmitis by five times [13]. This demonstrates the strong adherence of Moroccan surgeons to the European Society of Cataract and Refractive Surgeons (ESCRS) recommendations [14].

Nonsteroidal anti-inflammatory drugs (NSAIDs) have emerged as a crucial adjunctive tool. These drugs have been shown to reduce pain, control inflammation after surgery [15], and lower the risk of cystoid macular edema (CME) [16]. Despite all the advantages of these treatments, our survey showed that less than half of the respondents (46.5%) do not prescribe them extensively, unlike in the Korean and French surveys, in which 80% and 75.5%, respectively, prescribe them [4,11].

Limitations

This survey did not cover every ophthalmologist in Morocco. Some of the participants refused to respond to the online survey. Furthermore, cataract surgery is characterized by its unpredictability, and the surgeon's choices may vary based on the intraoperative situation and any potential complications that may arise. Most of the questions had single-response options, and surgeons were required to select only one response.

## Conclusions

This study provides some insight into the present cataract surgery techniques in Morocco, which might differ considerably from one country to another. Studies in various countries need to be undertaken to develop a consensus and reach some evidence-based patterns.
